# Functional Bowel Disorders in Iranian Population using Rome III Criteria

**DOI:** 10.4103/1319-3767.65183

**Published:** 2010-07

**Authors:** Majid Sorouri, Mohammad A. Pourhoseingholi, Mohsen Vahedi, Azadeh Safaee, Bijan Moghimi-Dehkordi, Asma Pourhoseingholi, Manijeh Habibi, Mohammad R. Zali

**Affiliations:** Research Center for Gastroenterology and Liver Diseases, Shahid Beheshti University (M.C), Tehran, Iran

**Keywords:** Functional bowel disorder, IBS, bloating, constipation, diarrhea, Rome III criteria

## Abstract

**Background/Aim:**

To study the prevalence and risk factors of functional bowel disorders (FBD) in Iranian community using Rome III criteria.

**Materials and Methods:**

This study was a cross-sectional household survey conducted from May 2006 to December 2007 in Tehran province, Iran, including 18,180 participants who were selected randomly and interviewed face-to-face by a validated questionnaire based on Rome III criteria.

**Results:**

In all, 1.1% met the Rome III criteria for irritable bowel syndrome (IBS), 2.4% for functional constipation (FC), and 10.9% of the participants had any type of FBD. Among participants with functional dyspepsia, 83.8% had FBD; the majority cases were unspecified functional bowel disorder (U-FBD). Of the subjects fulfilling the IBS criteria, IBS with constipation (52%) was the most frequent subtype. In the multivariate analysis, women had a higher risk of any FBDs than men, except for functional diarrhea (FD). The prevalence of FBD, FC and FD increased and IBS decreased with increasing age. Marital status was only associated with a decrease in the risk of FBD and FD, respectively. IBS subtypes compared with FC and FD. There was no significant difference between FC and IBS with constipation (IBS-C), except for self-reported constipation; while, IBS with diarrhea (IBS-D) had more symptoms than FD.

**Conclusion:**

This study revealed a low rate of FBDs among the urban population of Tehran province. The ROME III criteria itself, and the problems with interpretation of the data collection tool may have contributed in underestimating the prevalence of FBD. In addition the reliability of recall over 6 months in Rome III criteria is questionable for our population.

Functional bowel disorders (FBDs) include the irritable bowel syndrome (IBS), functional bloating (FB), functional constipation (FC), functional diarrhea (FD), and unspecified FBD (U-FBD) attributed to the small bowel, colon, and rectum. Although symptoms (e.g. diarrhea, constipation, bloating, pain) may overlap across these disorders, IBS is more specifically defined as pain associated with change in bowel habit, and this is distinct from FD and constipation characterized by change in bowel habit and no pain, or functional bloating when there is no change in bowel habit.[1,2] According to the ROME III criteria (the latest symptom-based diagnostic criteria of functional gastrointestinal disorders), onset of symptoms should begin at least 6 months before clinical presentation and the diagnostic criteria must be fulfilled for the last 3 months.[1,2] This time frame is less restrictive than Rome II (12 weeks of symptoms over 12 months).

The estimated prevalence of FBD and its subtypes varies enormously depending on the diagnostic criteria employed. Considering Rome II criteria, FBD has prevalence between 12.1 and 41.6% in different populations, and it is more frequent in women than in men.[3,4] In Iran, the prevalence of FBD was 40.1% in patients referred to a gastroenterology clinic.[5]

Of all FBDs, IBS has received more attention and its prevalence is 3-25%.[6–8] There are similar frequencies for IBS in Western countries, but may be lower in Asian countries and in African-American populations; however, there is a wide variation, even within individual countries.[1,7–9] In Iran, the prevalence of IBS was reported to be 5.8% in the general population and 3-18.4% in some groups.[10,5,11,12] While in the Rome II criteria, IBS was divided into three subtypes, in the Rome III criteria, it has four subtypes based on stool consistency alone: IBS with constipation (IBS-C), IBS with diarrhea (IBS-D), mixed IBS (IBS-M), and unsubtyped IBS (IBS-U).[2] It seems that IBS with an alternating stool pattern (IBS-A) (based on Rome II) maybe the most frequent IBS in Western countries, but in Asian countries there is no homogeneity.[11,13–16]

After IBS, FC seems to be the other more studied FBD. FC is a FBD that presents as persistently difficult, infrequent, or seemingly incomplete defecation, which does not meet IBS criteria. Constipation occurs in up to 27% of people depending on demographic factors, sampling, and definition. It affects all age groups and is most common in women and non-whites.[2,6]

In Iran, the prevalence of FC is obscure in the general population but in some groups, it was 3.1-28%.[5,12]

As for the other types of FBD, less data are available. Functional bloating (FB) is a recurrent sensation of abdominal distention that may or may not be associated with measurable distention, but is not part of another functional bowel or gastroduodenal disorder.[2] Up to 96% of IBS patients report this symptom and 10-30% of individuals in the general population report bloating during the previous year.[2]

The prevalence of FB was reported from 5.8% to 17.7%,[3,4,17] while the prevalence of FD was estimated from 0.4% to 9.6%.[3,18,19]

This paper aims to provide preliminary data on relative distribution of different types of FBD and their socio-demographic patterns based on a large sample of the general population, in Iran.

## MATERIALS AND METHODS

This study was part of a cross-sectional household survey conducted from May 2006 to December 2007 in Tehran province, Iran, which aimed to find the prevalence of gastrointestinal symptoms[20,21] and functional disorders[22–24] in Iranian community. A total of 18,180 adult persons drawn up randomly on the basis of the list of postal codes and random samples of these postal codes and their corresponding related address were drawn from the databank registry of Tehran central post office (approximately 5000 households selected and all members surveyed). These samples covered five cities including Tehran metropolitan, Damavand, Varamin, Firoozkouh, Pakdasht, and their rural constituencies. The sampled population was interviewed by trained health care workers at their own residence area. The research protocol was approved by the Ethics Committee of Research Center for Gastroenterology and Liver Diseases, Shaheed Beheshti Medical University, and all persons who participated in the study signed a consent form.

The questionnaire included two parts, with the first part containing data regarding personal and family characteristics (such as age, sex, educational level), which were recorded from every participant in the first place. In addition, participants were informed and asked about 11 gastrointestinal (GI) symptoms including abdominal pain/discomfort, constipation, diarrhea, bloating, heartburn/acid regurgitation, proctalgia, nausea/vomiting, fecal incontinence, bloody or black stool (melena), anorexia/weight loss, and difficulty of swallowing.

Participants who reported any of the above symptoms were referred for participating in the second interview by physicians in the vicinity. The second part of questionnaire consisted of questions about different gastrointestinal disorders, characterized on the basis of Rome III criteria.[4,5] The section of Rome III criteria was standardized in Persian designed by a working group, translated from English to Persian.

The validity and reliability of the Persian questionnaire was tested in a pilot study on 400 participants from city of Damavand. For validity study, the language, content, concurrence, and construct validities were examined. The test-retest reliability was good and the Cronbach alpha coefficient values were above 0.7 for all major symptoms included in the tool. Minor corrections, however, were made regarding some symptoms.[22–24]

Some demographic and clinical variables including sex (male/female), age, marital status (single, married, widow), education, and body mass index (BMI), were included in the analysis. The response rate for the first and second interviews was more than 92%, respectively.

All statistical analysis carried out using SAS version 9.1 (SAS Institute Inc., Cary, NC, USA). Pearson’s chi-square, contingency tables and logistic regression were performed to test for independence between discrete variables. Continuous variables are presented as mean±standard deviation and other parameters as frequency and percentage. A *P* value of 0.05 or less was considered statistically significant and all reported *P* values were two sided.

## RESULTS

A total of 18,180 entered in this cross-sectional study. The response rate was more than 92% and those who refused to participate in the interview were replaced with additional random samples. Among these participants 9072 (49.9%) were women. The mean age of men and women was 38.9±17.4 and 38.4±16.7 years (*P*< 0.001). Most participants were in age group of 16-29 years in both male and female groups.

In all, 1.1% met the Rome III criteria for IBS, 1.5% for FB, 2.4% for FC, and 10.9% of the participants had any type of FBD [Table 1]. Interestingly, 8.9% participants had functional dyspepsia and 83.8% of them had FBD; the majority cases with overlap of these were U-FBD [Table 1]. All FBDs were more frequent in female and divorced participants. The mean of age was higher in FBDs than healthy participants and the prevalence of FBD, FC and FB increased with increasing age.

**Table 1:** The prevalence of FBDs according to the Rome III criteria by sociodemographic characteristics and symptoms

	**FBD (%)§**	**IBS (%)**	**FC (%)**	**FD (%)**	**FB (%)**	**U-FBD (%)**	**Total N (%)**
Frequency	10.9	1.1	2.4	0.2	1.5	5.5	18180
Sex							
Male	7.8	0.6	1.2	0.2	1.4	4.3	9108 (50.1)
Female	13.7	1.5	3.7	0.3	1.7	6.7	9072 (49.9)
Age							
<40	6.5	0.6	1.4	0.2	0.9	3.3	12235 (67.3)
40-60	18.6	1.9	4.7	0.7	2.8	8.6	3962 (21.8)
>60	22.57	1.1	4.9	0.6	3.5	12.6	1980 (10.5)
Level of education							
Illiterate	12.2	0.7	2.9	0.4	1.4	6.8	4763 (26.2)
Below diploma	9.3	0.9	2.1	0.4	1.1	4.8	6690 (36.8)
High school diploma	11	0.9	2.5	0.4	2.1	5	4072 (22.4)
University education	11.1	1.1	2.1	0.5	2.6	5	2290 (12.6)
Masters or higher	10.5	1.1	1.6	0	0.5	7.1	360 (2)
Marital status							
Married	15.9	1.5	3.5	0.5	2.3	8.2	9362 (51.5)
Never married	3.6	0.2	0.7	0.2	0.5	1.9	8000 (44)
Widowed	25.7	1.1	8.8	0.6	3.4	12.7	590 (3.3)
Divorced	31.9	2.8	12.5	2.8	5.5	8.3	72 (0.4)
Abdominal pain	72.9	8.4	15.5	0.6	8.7	39.5	1196 (6.5)
Constipation†	74.5	7.6	31	2.3	6.8	27.2	1145 (6.3)
Diarrhea†	78.6	11.5	5.1	11.1	11.5	41.3	252 (1.4)
Bloating†	77.2	6.2	15.7	2.2	13.4	40.5	1610 (8.8)
Heartburn†	69.4	5.8	13	2	8.3	40.7	1584 (8.7)
Nausea/vomiting	68	10.9	15.5	2.5	6.7	32.8	238 (1.3)
Weight Loss†	62.9	8.9	15.6	1.9	8.6	28.6	315 (1.7)
Dysphagia	70.6	7.2	22.2	2	8.5	31.4	153 (0.8)
Fecal incontinence†	64.8	14.8	9.3	1.9	11.1	27.8	54 (0.3)
Functional Dyspepsia¶	83.8	8.1	17	1.2	-	57.5	1621 (8.9)

§ (%) Indicates the prevalence of FBDs subjects in that group.

† Self-reported symptoms.

¶ Functional dyspepsia is defined based on Rome III criteria.

FBD: Functional bowel disorder; FC: Functional constipation; FD: Functional diarrhea; FB: Functional bloating; U-FBD: Unspecified functional bowel disorder.

Self-reported constipation was a common symptom in FBDs. Its prevalence was 38.2%, 43%, 50.6%, and 77.5% in FD, FBD, IBS and FC, respectively. On the other hand, among participants with self-reported constipation, the proportion of FBD was 74.5% and that of FD, FC, IBS, and IBS-C was 2.4%, 31%, 7.6%, and 6.8%, respectively. Self- reported diarrhea was less common symptom (10%) in FBD than constipation, bloating, abdominal pain, and heartburn. Among participants with self-reported diarrhea, the proportion of FBD, FD, IBS, and IBS-D was 78.6%, 11.1%, 11.5%, and 5.2%, respectively.

The prevalence of self-reported bloating was 8.8% and in U-FBD patients, bloating was the most frequent symptom (64.4%).

IBS had the highest prevalence of abdominal pain (58.7%), nausea/vomiting (15.1%), weight loss (16.3%), depression (68%), anxiety (84.3%), and incontinence (4.7%) than other FBDs. Of the subjects fulfilling the IBS criteria, 45.3% were IBS-C; 20.3%, 19.2%, and13.4% were mixed IBS (IBS-M), unsubtyped IBS (IBS-U), and IBS with diarrhea (IBS-D), respectively. All subtypes of IBS increased with increasing BMI and except IBS-D, all were more frequent in women than men. It seems that IBS-D had the most symptoms [Tables 1 and 2]. In U-FBD, 64.4% reported bloating, 46.7% abdominal pain, 30.7% constipation, and 10.3% diarrhea.

**Table 2:** Prevalence of sociodemographic characteristics and symptoms in the different subtypes of IBS

	**IBS-C**	**IBS-D**	**IBS-M**	**IBS-U**
Frequency N (%)	78 (45.3)	23 (13.4)	35 (20.3)	33 (19.2)
Age (Mean±SD)	42.1±15.7	42.7±17.6	44.2±14	38.4±15.7
Sex§				
Male	17.9	52.2	31.4	33.3
Female	76.9	47.8	68.6	66.7
BMI				
< 25	47.4	34.7	25.7	33.3
>25	42.3	60.9	88.6	57.6
Level of education				
Illiterate	26.9	13	31.4	15.2
Below diploma	33.3	34.8	34.3	42.4
High school diploma	21.8	26.1	17.1	36.4
University education	17.9	21.7	14.3	3
Master or higher	-	4.3	2.9	3
Marital status				
Married	75.6	82.6	91.4	81.8
Never married	14.1	13	5.7	18.2
Widowed	6.4	4.3	2.9	-
Divorced	2.6	-	-	-
Abdominal pain	47.4	69.6	57.1	72.7
Constipation†	62.8	21.7	54.3	45.5
Diarrhea†	3.8	56.5	25.7	12.1
Bloating†	53.8	73.9	54.3	57.6
Heartburn†	42.3	69.6	57.1	60.6
Nausea/vomiting	7.7	30.4	22.9	12.1
Weight Loss†	12.8	30.4	17.1	15.2
Dysphagia	3.8	13	8.6	6.1
Fecal incontinence†	2.6	17.4	2.9	3
Depression†	69.2	60.9	68.6	69.7
Anxiety†	87.2	82.6	80	87.9
Functional dyspepsia¶	74.4	82.6	71.4	81.8

§ Proportion of sociodemographic characteristics and symptoms in the different subtypes of IBS reported as percent.

† Self-reported symptoms.

¶ Functional dyspepsia was defined based on Rome III criteria.

IBS-C: Irritable bowel syndrome with Constipation; IBS-D: Irritable bowel syndrome with diarrhea; IBS-M: IBS with mixed symptoms; IBS-U: Unsubtyped IBS

For the multiple logistic-regression analysis of risk factors associated with the FBDs, we incorporated age, sex, level of education, marital status, heartburn, abdominal pain, nausea/vomiting, anal pain, self-reported weight loss, dysphagia, fecal incontinence, anal bleeding, and self-reported constipation, diarrhea and bloating [Table 3]. Symptoms such as abdominal pain, heartburn and self-reported constipation, diarrhea, and bloating were the main predictors of FBDs. Women had a higher risk of any FBDs than men, except for FD. The prevalence of FBD, FC, and FD increased and IBS decreased with increasing age. While, marital status (only Never married and Widowed) was associated with a decrease in the risk of FBD and FD, respectively.

**Table 3:** Multiple logistic-regression analysis (final model) of whole cohort of participants, according to sociodemographic characteristics and symptoms§

	**FBD**	**IBS**	**FC**	**FD**	**FB**	**U-FBD**
Sex	1.42**	1.66**	1.83**	-	1.74**	1.19*
(reference, male)	(1.22-1.66)¶	(1.17-2.36)	(1.44-2.32)		(1.37-2.21)	(1.01-1.41)
Age (years)	1.07*	0.99*	1.01*	1.04**	-	1.01**
	(1-1.01)	(0.97-0.998)	(1-1.01)	(1.02-1.06)		(1-1.01)
History of abdominal surgery	-	-	0.66**	-	3.64	0.13**
			(0.52- 0.83)		(0.75-17.49)	(0.04-0.42)
Abdominal pain	6.21**	5.87**	1.85**	0.24**	1.87**	2.93**
	(5.07-7.6)	(3.85-8.96)	(1.43- 2.39)	(0.11-0.52)	(1.44-2.42)	(2.41-3.55)
Constipation†	12.49**	4.2**	28.27**	2.74**	27.82**	1.27*
	(10.24-15.24)	(2.89-6.1)	(21.66-36.92)	(1.49-5.05)	(21.32-36.3)	(1.03-1.56)
Diarrhea†	9.11**	2.17**	0.35**	30.77**	0.35**	2.36**
	(5.79-14.31)	(1.31-3.59)	(0.2- 0.61)	(16.27-58.22)	(0.2-0.62)	(1.68-3.31)
Bloating†	11.67**	1.9**	2**	2.11*	1.96**	6.1**
	(9.72-14)	(1.22-2.96)	(1.52-2.64)	(1.07-4.19)	(1.49-2.58)	(4.98-7.47)
Heartburn†	6.03**	1.49	1.53**	2.84**	1.53**	6.11**
	(5.01-7.27)	(0.98-2.28)	(1.17- 2)	(1.42-5.66)	(1.17-2)	(5.01-7.44)
Nausea/vomiting	-	1.98**	-	-	-	-
		(1.19-3.27)				
Fecal incontinence†	0.35*	-	0.34*	-	0.34*	0.44*
	(0.15-0.84)		(0.12-0.94 )		(0.12-0.94)	(0.22-0.9)
Anal bleeding†	-	-	1.55*(1.04- 2.32)	-	1.58*(1.06-2.37)	-

¶ Odds ratio and its 95%CI are reported.

† Self reported symptoms.

§ Age, sex, level of education, marital status, the history of abdominal surgery, heartburn, abdominal pain, nausea/vomiting, anal pain, dysphagia, fecal incontinence, and self-reported constipation, diarrhea, bloating, anal bleeding and weight loss were incorporated for the multiple logistic-regression analysis.

Data of marital status, weight loss, dysphagia, and anal pain are not presented. Marital status (only never married and widowed), level of education, and anal pain were only associated with a decrease in the risk of FBD, FD, and U-FBD, respectively, while weight loss and dysphagia had no association with any FBDs. FBD: Functional bowel disorder; FC: Functional constipation; FD: Functional diarrhea; FB: Functional bloating; U-FBD: Unspecified functional bowel disorder.

* *P*<0.05

** *P*<0.01

IBS subtypes compared with FC and FD. There was no significant difference between FC and IBS-C, except for self-reported constipation (77.5% vs. 62.8%; *P*< 0.00). While, abdominal pain, nausea/vomiting, weight loss, fecal incontinence and anal pain were more frequent in IBS-D than FD [Table 4].

**Table 4:** Comparison of sociodemographic data and symptoms between those with FD and those with IBS-D

	**IBS-D N (%)**	**FD N (%)**	***P* value**
Female	11 (47.8)	38 (55.9)	0.5
Age (Mean+SD)	42.7+17.6	45.5+18	0.78
Level of education			0.4
Illiterate	3 (13)	16 (23.5)	
Below diploma	8 (34.8)	24 (35.3)	
High school diploma	6 (26.1)	16 (23.5)	
University education	5 (21.7)	10 (14.7)	
Master or higher	1 (4.3)	0	
Marital status			0.59
Married	19 (82.6)	45 (66.2)	
Never married	3 (13)	15 (22.1)	
Widowed	1 (4.3)	4 (5.9)	
Divorced	0	2 (2.9)	
History of abdominal surgery	12 (52.2)	20 (29.4)	0.12
Abdominal pain	16 (69.6)	7 (10.3)	0.000
Constipation†	5 (21.7)	26 (38.2)	0.15
Diarrhea†	13(56.5)	28 (41.2)	0.2
Bloating†	17 (73.9)	35 (51.5)	0.06
Heartburn	16 (69.6)	33 (48.5)	0.08
Nausea/vomiting	7 (30.4)	6 (8.8)	0.01
Weight loss†	7 (30.4)	6 (8.8)	0.01
Dysphagia	3 (13)	3 (4.4)	0.15
Fecal incontinence†	4 (17.4)	1 (15)	0.004
Anal pain	6 (26.1)	2 (2.9)	0.001
Anal bleeding†	1(4.3)	3 (4.4)	0.99

† Self reported symptoms.

FD: Functional diarrhea; IBS-D: Irritable bowel syndrome with diarrhea

## DISCUSSION

Our findings suggest that the FBD is less common in general population than previously reported;[3,4] it was more frequent among women, and this was also true with all subtypes of FBD except FD. Our results confirms the study of Thompson *et al*. in Canada which showed higher prevalence of FBD in female[3] and other studies, including an Iranian population based, demonstrating higher prevalence of IBS and FC among women.[4–8,25–31] Nevertheless, some studies in Taiwan, India, and another Iranian study (among university students) reported the same prevalence of IBS among men and women.[11,32–35]

History of abdominal surgery was significant only in the models of FC and U-FBD. Similarly, having reviewed relative evidence on IBS only, a systematic review by Hasler and Schoenfeld states that there is still poor evidence to link IBS and a history of abdominal surgery.[36] But Roshandel *et al*. reported a high prevalence of abdominal surgery among FBD patients referred to gastroenterology clinics.[5]

This large population-based survey demonstrates a community prevalence of IBS of 1.1% (0.6% of men and 1.5% of women). Although the Rome criteria are simplified in the 3rd version, our estimate of the prevalence of IBS is one of the lowest that has been reported. In Turkey, the overall prevalence of IBS was 6.3% while a similar rate of 4.1% was found in a study conducted in Hong Kong among ethnic Chinese subjects.[37,38] In Iran, the prevalence of IBS was reported to be 5.8% in the general population and 3-18.4% in some groups.[5,10–12] Our finding indicated low prevalence for IBS. As one possibility, the prevalence estimates may vary because of the specific questions used to elicit the information. Careful interpretation of the abdominal discomfort or pain and stool characteristics is the most important step in recognizing IBS. In this study, face-to-face interview was adopted and all subjects were requested to fill in a questionnaire assisted by trained health personnel who could provide the relatively precise interpretation of the items in the questionnaire. Another problem is in the recall nature of the data in asking the Rome III criteria questions we used. It is surely difficult for anyone to remember precisely whether they had abdominal pain over the past 6 months, unless their symptom frequency lies near either extreme of these ranges.

Our finding of a somewhat lower age for IBS patients is compatible with previous studies,[28,30,33] although two studies in China and England found no relationship between IBS and age.[16,39]

Of the subjects fulfilling the IBS diagnostic criteria in our study, the majority of cases were IBS-C (45.3%), while the cases with IBS-D (13.4%) were the least common. Together IBS-M and IBS-U comprised of 39.5% cases. An international survey of 40,000 subjects across eight industrialized European countries by Hungin *et al*. also revealed IBS-A (63%) to be the most frequent subtype, followed by IBS-D (21%) and IBS-C (16%).[13] Another survey in the USA by the same author and using the same methodology showed that IBS-A comprised the majority (66%) of IBS cases, followed by IBS-D (21.3%) and IBS-C (12.7%).[14] In contrast, a Spanish study on 2000 subjects showed that IBS-C (37%) was the most common subtype among 63 subjects meeting Rome II, followed by IBS-D (25%) and IBS-A (23%).[40] In Asia, a study in Malaysia also showed that IBS-C was the most frequent subtype (77.4%), and IBS-A and IBS-D comprised 15.5 and 7.1% of IBS patients, respectively.[15] In contrast to our study, Xiong *et al*. reported IBS-D to be the most frequent (74.1%) and IBS-A as the least frequent (10.8%) subtype in China.[16] In Iran, among IBS patients referred to gastroenterology clinic, Roshandel *et al*. reported IBS-A as the most frequent (60%) and IBS-C and IBS-D to be 29.1% and 10.9%, respectively. Interestingly, another Iranian study on 1200 university students from western Iran reported IBS-C (50%) as the most frequent and IBS-A (21%) as the least frequent subtype (with IBS-D comprising 29% of IBS cases).[11] It seems that IBS-A maybe the most frequent IBS in Western countries, but in Asian countries there is no homogeneity.

Advancing age and female gender were independently associated with a diagnosis of FC in our study. Talley *et al*. did not find gender to be associated with FC;[41] this differs from most other studies, which have found FC rates to be higher in women.[42–44] Like ours, Sandler *et al*.’s study in the United States found no association between FC and education.[43] The association of FC with advancing age concurs with other studies.[28,42,43,45–47]

To date, it seems that most studies have focused on IBS and FC, and other FBDs as defined by Rome criteria have been investigated less. In two studies in Israel and one study in Canada, the prevalence of FB was reported to be 5.8-17.7%;[3,4,17] but in our study, the prevalence of FB was as low as 1.5%. Our results confirm the study of Thompson *et al*. in Canada which showed higher prevalence of FB in females.[3]

In all participants, 8.8% of individuals reported bloating during the previous 6 months, but it was 10-30% based on Rome II in some community surveys.[48,49]

In our study, FD had a low prevalence. It was reported by 9.6% of Minnesota residents[18] and 4.8% and 8.5% of people throughout the United States and Canada, respectively.[3,19] On the other hand, an Israeli study on 981 individuals showed that 0.4% had FD.[4] In Iran, the prevalence of FD was reported to be 2% in 1023 gastroenterology outpatients.[5]

In U-FBD patients, bloating was the most frequent symptom (64.4%) and 46.7% of them reported abdominal pain. Interestingly, functional dyspepsia (based on Rome III criteria) coexisted in 92% of U-FBD. In Rome criteria, coexistence of the symptom of bloating and any functional GI disorder excludes FB and patient will be categorized as U-FBD. Therefore, it seems that a majority of U-FBD patients may have been those who did not fulfill IBS or FB criteria.

To our knowledge, because patients were sampled from a general population, the selection biases that might apply to a specialist or hospital derived sample could not arise. Although all of participants live in Tehran province and our data may not represent the entire Iranian population, the large sample size serves as a strength of the study.

In conclusion, this study revealed a low rate of FBDs among the urban population of Tehran province. Our experience indicated that the ROME III criteria itself, and the problems with interpretation of the data collection tool might have taken part in underestimating the prevalence of FBD. In addition the reliability of recall over 6 months in ROME III is questionable for our population.
